# Modeling Chinese Teachers’ Efficacies for the Teaching of Integrated STEM With Interdisciplinary Communication and Epistemic Fluency

**DOI:** 10.3389/fpsyg.2022.908421

**Published:** 2022-06-02

**Authors:** Pei-Yi Lin, Ching Sing Chai, Weifeng Di, Xingwei Wang

**Affiliations:** ^1^Department of Education and Learning Technology, National Tsing Hua University, Hsinchu, Taiwan; ^2^Department of Curriculum and Instruction, The Chinese University of Hong Kong, Hong Kong, Hong Kong SAR, China; ^3^Key Laboratory of Intelligent Education Technology and Application of Zhejiang Province, Zhejiang Normal University, Jinhua, China; ^4^College of Teacher Education, Zhejiang Normal University, Jinhua, China; ^5^College of Control Science and Engineering, China University of Petroleum, Qingdao, Shandong Province, China

**Keywords:** science technology engineering mathematics education, interdisciplinary communication, epistemic fluency, teacher efficacy, technological pedagogical content knowledge

## Abstract

Engineering design is a core activity in integrated science, technology, engineering, and mathematics (STEM) education. During the design process, teachers should possess interdisciplinary communication capacities to collaborate with their peers who are teaching different subjects and have epistemic fluency to comprehend multiple ways of subject matter knowing for the collective design of high-quality integrated STEM (iSTEM) lessons. This is especially so for the online mode of instruction during and after the pandemic. Teachers’ efficacies for interdisciplinary communication and epistemic fluency have rarely been explored. In this study, we aimed to examine primary school, junior high school, and high school STEM teachers’ (N = 483) efficacies for daily instruction, student engagement, interdisciplinary communication, epistemic fluency, and technological pedagogical engineering knowledge (TPEK) and designing integrated STEM instruction. An exploratory factor analysis (EFA) (*n* = 155) and a subsequent confirmatory factor analysis (CFA) (*n* = 328) were used to validate the measurement and structural model. Next, a structural equation model (SEM) was employed to examine whether these variables were reliable predictors of teachers’ integrated STEM instruction. The survey was validated with good reliabilities and the structural equation modeling supported most of the hypotheses. Statistically, the results also showed that teachers’ general efficacies for daily teaching and students’ engagement predicted their interdisciplinary communication, epistemic fluency, and TPEK. Teachers’ interdisciplinary communication predicted their epistemic fluency, TPEK, and iSTEM. Teachers’ epistemic fluency also predicted their TPEK and iSTEM. In addition, multi-group analyses were used to test the measurement invariance of the scale and to compare the latent means between the genders and subject matters. The results of the various analyses confirmed that the measurement model appeared to be equivalent across the genders and subject matters examined. Genders and subject matters did not significantly differ in any of the measured variables. The results from this study indicate that teachers’ epistemic fluency and interdisciplinary communication play essential roles in advancing their TPEK and iSTEM. Hence, this study suggests that teacher professional development should focus on enhancing teacher epistemic fluency through interdisciplinary collaboration to support the development of TPEK and iSTEM instruction.

## Introduction

There has been increasing emphasis on incorporating integrated science, technology, engineering, and mathematics (STEM) curricula into K-12 education because it advances a country’s economic competitiveness (Kelley and Knowles, 2016; Hoeg and Bencze, 2017). All citizens, including both STEM and non-STEM professionals, should possess STEM literacy to have a deeper understanding of STEM domains and to apply their STEM understanding to resolve problematic situations in their lives (National Research Council, 2014). The use of integrated STEM instruction is a promising approach to improving students’ STEM learning. Research on STEM educational reform has been focusing on students’ STEM learning process (Zheng et al., 2020), outcomes (Wahono et al., 2020), and its relation to their future career pursuits (Reinhold et al., 2018) with some positive results. This has also led to the demands for teachers’ competencies for disciplinary integration (Wu et al., 2019; Chiu et al., 2021). In addition, pedagogically sound use of technologies, including using online collaborative platforms, is essential to facilitate students’ STEM learning beyond the constraints of classroom time and space. Moreover, the nature of STEM learning necessarily involves technologies such as productivity and ideation tools (such as using computer-assisted design software), and communication tools to manage emerging ideas (Chai et al., 2020). Teachers’ competencies in these aspects are generally referred to as their technological pedagogical content knowledge (TPACK; Chai et al., 2019). With strong TPACK, teachers are generally able to leverage the online and face-to-face mode of instruction, hence reducing the effects created by incidents such as the COVID-19 pandemic.

However, traditional teacher training isolates each discipline, and teachers lack experience in designing an interdisciplinary curriculum that synthesizes various types of discipline knowledge and implementing integrated curricula in the classroom (Ryu et al., 2019; Wu et al., 2019; Cunningham et al., 2020). Teachers’ efficacy for designing integrated and interdisciplinary STEM learning has been examined (DeCoito and Myszkal, 2018; Chai, 2019; Pressley and Ha, 2021). These studies also indicated that teachers’ efficacy for designing STEM learning is associated with their disposition toward designing STEM learning activities and participating in designing STEM activities (DeCoito and Myszkal, 2018; Lin et al., 2021). Nonetheless, the specific capacities contributing to integrated STEM lesson designs need further exploration (Chai, 2019).

Teachers’ design thinking has been identified as a key competency to develop an integrated STEM curriculum (see, e.g., Nguyen and Bower, 2018; Wu et al., 2019; Chiu et al., 2021). Situated in the interdisciplinary context and the task of integrating the subject matters, it seems obvious that STEM teachers need to traverse the disciplinary boundary. Epistemic fluency refers to one’s ability to recognize, appreciate, and engage in interdisciplinary design discourses to collaboratively create new knowledge and practices that can address emerging problems (Goodyear and Zenios, 2007). Science, engineering, and mathematics are undergirded by distinct forms of epistemologies. When teachers design iSTEM, they need to engage in different ways of thinking and knowing (Markauskaite and Goodyear, 2017). Therefore, it is necessary to further develop teachers’ epistemic fluency for fruitful interdisciplinary STEM collaboration in pedagogical design work.

Particularly, the use of engineering design in STEM instructional strategies has become the main approach to implementing iSTEM curricula (Capobianco et al., 2018; Chai, 2019; Lee et al., 2019). Many studies have also confirmed that engineering design entails complex problem-solving processes that require students to use and integrate scientific, mathematical, engineering, and technological knowledge to design the process or product (Fan and Yu, 2017; Park et al., 2018; Aranda et al., 2020). Technologies are not only communication tools that support students’ participation in learning (de Jong, 2019). They can also be designed to provide adaptive scaffolding, serve as an online platform to support knowledge and idea-sharing and negotiation beyond class time, and document the emerging learning outcomes when solving engineering problems (Dasgupta et al., 2019; Wang and Chiang, 2020). The pedagogical use of technologies to facilitate students’ integration of science and mathematics knowledge to resolve engineering design challenges is referred to as the teachers’ technological pedagogical engineering knowledge (TPEK; Chai et al., 2019). Teachers’ TPEK has recently been reported to be positively associated with the teachers’ efficacy for iSTEM.

Existing STEM research mainly focuses on the implementation of iSTEM curricula, but these studies have not fully investigated the articulation and integration of distinctive subject matter knowledge and epistemologies of practices (Reynante et al., 2020). Hence, this study proposes an iSTEM framework that includes teachers’ efficacy for daily teaching and student engagement, epistemic fluency, TPEK, and iSTEM. In addition, we examined how teachers’ efficacies in these areas are interrelated to provide educators with references that could facilitate teacher development.

## Literature review

**Figure 1:**
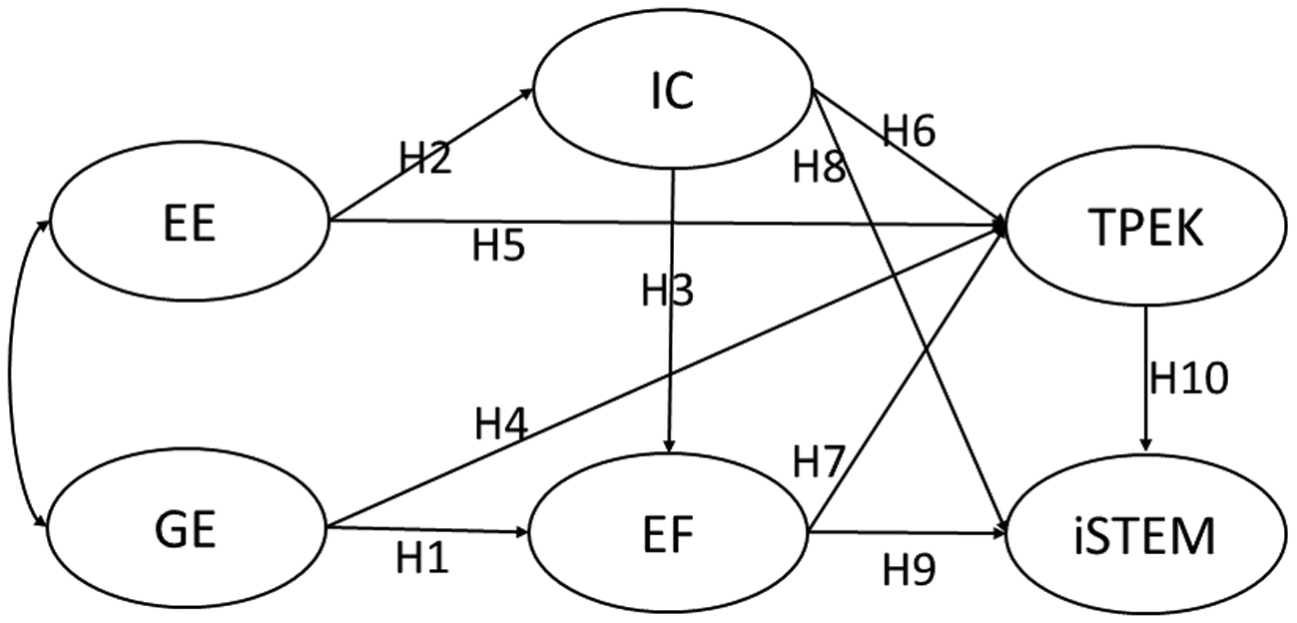
The hypothesized model.

As indicated, teachers face challenges when designing integrative STEM instruction because it involves interdisciplinary curriculum integration. To be an effective teacher to teach subject matter knowledge, two of the important forms of efficacies all teachers need to perform their duties are general efficacy in subject matter teaching and their efficacy to engage students (Tschannen-Moran et al., 1998). To design and implement the iSTEM curriculum, teachers would need to build on these efficacies to develop interdisciplinary communication, epistemic fluency, and TPEK, to finally acquire the complex set of competencies for iSTEM. Hence, this study reviewed the relevant teachers’ efficacies for designing iSTEM to formulate the hypotheses (H; see Figure 1).

### Teachers’ Self-Efficacy

Self-efficacy reflects a person’s expectation of success when carrying out a certain task (Bandura, 1986; Bong and Skaalvik, 2003). Teacher self-efficacy refers to “the teacher’s belief in his or her capability to organize and execute courses of action required to accomplish a specific teaching task in a particular context” (Tschannen-Moran et al., 1998, p. 233). Two important constructs that are commonly used to measure teacher efficacies are their general teaching efficacy (GE) to use teaching skills to deliver effective instruction and their efficacy to engage students in learning activities (EE; Tschannen-Moran and Hoy, 2001; Yin et al., 2017). Teachers with higher self-efficacy believe that they are able to use effective instructional strategies and engage students in learning (Tschannen-Moran et al., 1998; Künsting et al., 2016; Fackler et al., 2021). Previous studies have also indicated that teachers’ self-efficacy is related to the generation of new teaching ideas (Ross and Bruce, 2007; Lin et al., 2021), implementation of alternative teaching strategies to address students’ needs (Fackler et al., 2021; Pressley and Ha, 2021), greater efforts to foster thinking skills (e.g., creative thinking and critical thinking), and motivating students (Dilekli and Tezci, 2016; Burić and Kim, 2020). Thus, this study considers teachers’ self-efficacy as being related to their general teaching effectiveness and student engagement.

STEM learning is different from traditional learning which positions students as passive learners (Struyf et al., 2019). Many integrated STEM teaching practices emphasize students’ capacities to use scientific or mathematical ideas in engineering or technological design contexts (e.g., So et al., 2018; Wilson, 2020; Chang and Yen, 2021). Students need support to elicit the relevant disciplinary knowledge for the integrated contexts. Hence, STEM teachers need to design well-integrated instruction that provides opportunities for students to learn in more relevant and stimulating contexts and encourages the use of integrative STEM knowledge (Kelley and Knowles, 2016).

Previous studies have indicated that STEM teachers with high self-efficacy can collaborate on curriculum design and effectively implement integrated STEM knowledge and learning activities (Boschman et al., 2015; Chiu et al., 2021). Given the reviewed studies about teachers’ efficacy in relation to creating new and alternative teaching and learning activities, we hypothesized that STEM teachers with high self-efficacy (represented by general instructional efficacy, GE, and engagement efficacy, EE) are more likely to develop an interdisciplinary understanding of how different STEM knowledge works in the classroom, and communicate and collaborate with other subject teachers in the creation of STEM knowledge and practices. In particular, teachers’ GE should predict their epistemic fluency (see next section). The former denotes flexible use of pedagogical knowledge, while the latter involves flexible use of multiple discipline knowledge. Teachers’ GE is likely to be more established, and their epistemic fluency is an emerging competency as teachers engaged in designing and implementing STEM projects. Hence, H1 is formulated. Similarly, teachers’ GE should predict their technological pedagogical engineering knowledge (TPEK; i.e., H4) as the latter is a specific form of expertise. In addition, teachers’ EE is about how teachers engage students in general. Facilitating students’ engagement necessarily involves communicating with students (Struyf et al., 2019). It requires teachers to inquire about students’ progress, the challenges students face, their doubts, and difficulties, and suggesting alternative approaches, modeling creative problem solving, and provide emotional support. In other words, engagement efficacy is founded on communication skills. It is necessary for teachers to have such skills to facilitate learning. Interdisciplinary communication could be viewed as a more complex form of competency involving adults working together with different ways of knowing, and at times could be competing for resources. Hence, teachers’ EE should be more fundamental to teachers’ interdisciplinary communication competency and teachers’ EE should predict IC (i.e., H2). Teachers’ EE should also predict their TPEK, as the latter is a specific form of teachers’ knowledge that engages students in work on engineering problems supported by technologies (i.e., H5).

### Epistemic Fluency and Interdisciplinary Communication

Epistemic fluency is defined as one’s ability “to identify and use different ways of knowing, to understand their different forms of expression and evaluation, and to take the perspective of others who are operating within a different epistemic framework” (Morrison and Collins, 1996, p. 109). It is reflected among knowledge workers who are flexible and adept at generating new insights and are able to construct actional knowledge in complex epistemic environments (Morrison and Collins, 1996; Goodyear and Ellis, 2007; Markauskaite and Goodyear, 2017), i.e., it is fundamental for the synthesis of knowledge to address real-life authentic problems that defy discipline-based approach. Previous studies have noted that epistemic fluency can be acquired through participation in collaborative knowledge work for the improvement of ideas (Goodyear and Zenios, 2007; i.e., H3).

Epistemic fluency has been highlighted as an enabler for the design of integrated STEM lessons (Chai, 2019). When teachers design integrative STEM projects, they need to identify appropriate authentic problems that will motivate students to seek and integrate knowledge from different disciplines to generate design ideas (Goodyear and Zenios, 2007; Nguyen and Bower, 2018). In other words, STEM teachers need epistemic fluency to understand diverse forms of knowledge related to the problems to design the integrated STEM projects which are represented by TPEK (i.e., H7).

Teachers usually possess expertise only in one or two subject matters. However, the design of integrated STEM projects usually involves teachers from different disciplines and at times engineering experts or professors (Chai, 2019). Teachers thus need to have the capacity to work with other professionals to generate integrative STEM knowledge and knowing and use different epistemic strategies to guide students in using and constructing subject matter knowledge (Markauskaite and Goodyear, 2017). Hence, when STEM teachers teaching different subject matter collaborate to design a STEM project, they need to negotiate a shared epistemological framework to avoid being narrowly focused on their own teaching experience and discipline knowledge (Reynante et al., 2020). Continuous negotiations would be needed to move teachers toward sharing their knowledge sources and epistemological frame of reference (Morrison and Collins, 1995; Mor and Abdu, 2018). This will ensure that the teachers could adopt others’ perspectives to understand different epistemic frameworks. The interdisciplinary communication (IC) competencies, therefore, undergird teachers’ EF development. In sum, previous studies indicate that epistemic fluency is acquired through the discourses in the collaborative construction and improvement of STEM teaching ideas (Goodyear and Zenios, 2007; Chai et al., 2020). Hence, H3 is formulated. In a similar vein, i.e., interdisciplinary communication facilitates interdisciplinary understanding which equips teachers with richer STEM knowledge, IC should also predict the teachers’ integrative STEM (iSTEM) efficacy (i.e., H8).

### Teachers’ Technological Pedagogical Engineering Knowledge for iSTEM Practices

STEM education has garnered increasing attention because we are facing a world with complex problems. To solve these real-world problems, we should possess multiple types of knowledge, concepts, and skills that ground the integration of STEM disciplines (Bybee, 2010). However, teachers encounter many epistemic challenges when designing integrated STEM (Chai, 2019; Wu et al., 2019), particularly, when they connect individual discipline understanding to relevant disciplines and real-world contexts (e.g., Moore et al., 2014; Kelley and Knowles, 2016; Roehrig et al., 2021). Reviews of previous studies mainly focused on science and mathematics rather than on interdisciplinary STEM teaching and learning (Bell, 2016; English, 2016; Kelley and Knowles, 2016). Engineering design and its application using technology to solve real-world problems are increasingly emphasized and employed (e.g., Capobianco et al., 2018; Park et al., 2018; Aranda et al., 2020), teachers need to acquire engineering knowledge and convert it into a pedagogical design represented in the form of integrated STEM projects. There is a well-documented need for teacher professional development for STEM education (Margot and Kettler, 2019; Ryu et al., 2019; Roehrig et al., 2021). Therefore, this study investigates a teacher’s capacities to design and integrate technological knowledge, engineering design knowledge, and pedagogical knowledge in iSTEM curriculum and instruction. It is a specialized type of efficacy that iSTEM teachers should possess.

Technology is not as clearly defined as a discipline such as science, mathematics, and engineering (McComas and Burgin, 2020), but it is increasingly integrated into iSTEM learning (Lee et al., 2020; Wang and Chiang, 2020). Current integrative STEM projects necessarily involve the use of engineering design and technology for collaborative ideation, making and coding research, and testing of prototypes to solve real-world problems (e.g., Chang and Chen, 2020; Fidai et al., 2020). In other words, technologies play multiple facilitative roles as tools for sense-making, documentation, and production. Integrating technology into instruction is a complex and challenging process, for a single subject matter (Chai, 2019). In the integrative STEM context, the epistemic challenges are further aggravated since each discipline is supported by different technologies associated with different pedagogies (e.g., simulation for science inquiry), which need to be coordinated for teachers’ design of iSTEM learning (Capobianco et al., 2018; Nguyen and Bower, 2018; Cunningham et al., 2020). In addition, adequate preparation for the iSTEM curriculum faces multiple barriers such as time, communication, material, unclear curriculum standards, and teachers’ readiness (Shernoff et al., 2017; Margot and Kettler, 2019; Wang and Chiang, 2020).

In response to these challenges and barriers, it is necessary to engage teachers in epistemic activities to co-design with other professionals to create needed knowledge (Shernoff et al., 2017; Wu et al., 2019; Stammes et al., 2020). The interplay among the disciplines is complex and requires teachers to deliver the learning content purposefully and deliberately so that students can transfer their knowledge and skills in new or authentic contexts (Moore et al., 2014; Kelley and Knowles, 2016). Based on Mishra and Koehler’s (2006) technological pedagogical content knowledge (TPACK) model, teachers need to interweave technological pedagogical science, mathematics, and engineering knowledge to develop and design a TPACK-STEM curriculum (Chai et al., 2020). Recent research indicated that the teachers’ technological pedagogical science knowledge and mathematics knowledge are predictors of their technological pedagogical engineering knowledge (TPEK). Hence, TPEK can represent the form of complex knowledge that integrates teachers’ technological knowledge, engineering knowledge, and pedagogical knowledge, and it operationalizes the expertise of teaching integrated STEM lessons (Chai et al., 2019). As argued earlier, TPEK requires IC and EF to support it (i.e., H6 and H7). Chai et al. (2019) reported that TPEK is predictive of teachers’ integrative STEM efficacy (iSTEM) (i.e., H10). Since EF and IC should predict TPEK, they may also exert direct effects on iSTEM (i.e., H8 and H9).

In sum, this study examines iSTEM through the analytical identification of the various forms of competencies that the teachers need. Building on their daily teaching competencies (GE and EE), when teachers are involved in designing iSTEM curricula, they should generate a new understanding of each STEM discipline and multiple ways of subject matter knowing (EF) through interdisciplinary communication (IC). The co-designing helps teachers to construct their TPEK and their iSTEM, which ideally addresses the pedagogical challenge of integrative STEM education. The literature to date hence supports the construction of Figure 1, which depicts the teachers’ competencies for STEM education. The research questions formulated in this study to test the theoretical framework in Figure 1 are listed below:

1. Does the instrument designed to measure teachers’ perceptions of their teaching efficacies (i.e., general efficacy, GE, and engagement efficacy, EE), interdisciplinary communication (IC), epistemic fluency (EF), technological pedagogical engineering knowledge (TPEK), and teachers’ ability to integrate STEM (iSTEM) provide a reliable and valid factor structure?
2. Are the hypotheses of teachers’ efficacy for integrative STEM teaching supported?
3. Is the measurement invariant across participants of different genders or subject matters?
4. If measurement invariance is found, are there latent mean differences between the genders and subject matters with respect to the latent variables (i.e., GE, EE, IC, EF, TPEK, and iSTEM) in the teachers’ efficacy for integrative STEM teaching scales?

The hypotheses (see Figure 1) are H1: GE predicts EF; H2: EE predicts IC; H3: IC predicts EF; H4: GE predicts TPEK; H5: EE predicts TPEK; H6: IC predicts TPEK; H7: EF predicts TPEK; H8: IC predicts iSTEM; H9: EF predicts iSTEM; and H10: TPEK predicts iSTEM.

## Materials and Methods

### Participants

There were 483 teachers from China (male = 63.15%). The mean age of the teachers was 30.12 years (*SD* = 2.61 years). This was an advanced online training workshop, the participants had an average of 134.2 h of STEM training time (*SD* = 39.8 h), and the average STEM teaching experience was 15.4 months (*SD* = 3.58 months). The participants were elementary school (n = 215, 44.5%), junior high school (n = 223, 46.17%), and high school (n = 45, 9.32%) teachers. These teachers’ major teaching subjects are either physics (n = 104, 21.5%), mathematics (n = 91, 18.8%), information technology (n = 195, 40.4%), and science (n = 93, 19.3%) at schools. They were trained on using Tencent Meet for online teaching and a designated online platform for the students to run tests of their coding during their STEM lessons. They participated voluntarily with informed consent and completed the online self-reported survey after the online training. The sampling strategy is hence a mix of purposive (i.e., they had substantial STEM teaching experience) and convenience sampling.

This online training took place once a week, one hour each time, for a total of 12 hours. The fourth author was the instructor. The participants took the online course and they had to design Arduino microcontroller programming tasks and a prototype STEM teaching unit for their school. They use the prototype to lead their school interdisciplinary project team to finalize and implement the teaching unit. The Tencent Meeting supports instant interactions. The participants can ask questions at any time using a microphone or typing in the chatbox during the training sessions. They received online feedback to correct and verify their Arduino projects, and revise their designed syllabus by experienced teachers in terms of STEM skills (e.g., programming and engineering design). Additional support was provided through the WeChat group when the teachers faced problems.

### Instruments

The instrument measured six factors, namely, general efficacy, engagement efficacy, interdisciplinary communication, epistemic fluency, technological pedagogical engineering knowledge (TPEK), and integrated STEM curriculum (iSTEM). Participants self-reported their perceptions regarding the questionnaire items. The instruments for “general efficacy” (four items, α = 0.88; e.g., “I am able to use different teaching strategies in my classroom”) and “engagement efficacy” (four items, α = 0.88; e.g., “I can motivate students who show low interest in schoolwork”) were based on the survey items reported by Yin et al. (2017) as it has been tested among Chinese teachers. TPEK (six items, α = 0.89; e.g., “I am able to present real-world problems using technology (video-cases, web-based materials, etc.) to initiate an engineering project”) and iSTEM (five items, α = 0.94; e.g., “I am able to formulate good STEM problems to stimulate students interdisciplinary knowledge construction”) were based on the survey developed and validated by Chai et al. (2019).

The “interdisciplinary communication” items (six items, α = 0.90; e.g., “I can communicate with colleagues from other disciplines in the context of interdisciplinary cooperation”) and epistemic fluency (six items, α = 0.91, e.g., “I am able to use different discipline-based methods of knowledge construction (e.g., science, mathematics, and engineering) flexibly”) were developed specifically by the authors for this research based on Markauskaite and Goodyear’s (2017) extensive works on epistemic fluency. To establish face validity, the initial 17 items generated by the authors were subjected to two rounds of expert review. The first round of reviews was provided by four professors in educational technology. The items were revised and subjected to five other professors including four in educational technology and one in educational psychology. Finally, six items for each factor were retained for the survey.

All items were scored on a 5-point Likert scale (i.e., 1 = strongly disagree to 5 = strongly agree). The items are listed in the Appendix.

### Data Analysis

For the first and second research questions, an exploratory factor analysis (EFA) (*n* = 155; 65.2% male) and Cronbach’s alpha were reported to assess the internal consistency of the survey using the IBM Statistical Product and Service Solutions version 21. The sample was randomly selected by SPSS for 33% of the data, ensuring that the sample size for EFA with the general rule of 1 item to 5 participants. Then, a confirmatory factor analysis (CFA; *n* = 328; 62.2% male) was performed to validate the measurement model using AMOS 20. Mardia’s multivariate kurtosis coefficient was adopted to assess the multivariate normality of the measurement model (Mardia, 1980). The Mardia’s multivariate kurtosis coefficient value obtained in this study was 11.613, which is less than the recommended value of p (p + 2) = 31(33) = 1,023 (where p is the number of observed variables). Thus, the model’s multivariate normality was satisfied. The construct validity and the survey constructs (i.e., the analysis of convergent validity and the analysis of discriminant validity) were assessed. The evaluation of the model fit was then performed to verify the adequacy of the constructs. Next, path analysis was conducted to test the hypotheses proposed in this study. After validating the survey, multi-group invariance analyses were conducted to compare group differences using AMOS 20.0 to answer the third and fourth research questions. The multi-group analysis was employed to test the invariance of the measurement model (i.e., configural, metric, and scalar invariance tests) across groups before we made a comparison of the sub-groups to assess if there were significant differences in the latent variables (Hong et al., 2003). Measurement invariance indicated that the hypothesized model has the same meaning under different conditions. Otherwise, latent mean differences might not reflect differences in the latent construct.

## Results

### Factor Analysis of the Survey

In the preliminary analysis, an exploratory factor analysis (EFA) (*n* = 155) was carried out using principal axis factoring analysis and the direct oblimin rotation technique to clarify the factor structure of the survey. Factors with an eigenvalue greater than 1 were retained, and this was supported by the scree plot. The factor analysis outcomes indicated that the measures of sampling adequacy were acceptable: all loadings were above 0.5, the Kaiser–Meyer–Olkin measure of sampling adequacy was 0.92, and Bartlett’s test of sphericity was significant χ2 (465) = 3443.871 (*p* < 0.001). The final factor structure identified six factors that reflect the distinctions among the teachers’ efficacies in designing interdisciplinary STEM teaching. The factors explained 72.60% of variances. Each factor was named according to the loaded items: general efficacy (0.79–0.89, explained 9.84% variance); engagement efficacy (0.77–0.87, explained 5.77% variance); interdisciplinary communication (0.64–0.86, explained 4.02% variance); epistemic fluency (0.78–0.80, explained 39.66% variance); TPEK (0.71–0.81, explained 7.44% variance); and integrative STEM (0.82–0.87, explained 5.88% variance). The overall α was 0.95. All constructs had acceptable reliability. According to Kline (2005), non-normality is defined as a skewness value greater than 3 and a kurtosis value greater than 10. The skewness and kurtosis values are below the thresholds (see Table 1) and could be regarded as fairly normal for further analyses. We also looked into the correlation among the variables. Pearson’s correlations were computed for the primary sample (*n* = 155) to take a look at the relationships among the variables, which indicated that significant and positive relationships exist among all of them (from *r* = 0.26 to *r* = 0.66, *p* < 0.01). This suggests the existence of significant positive correlations among these factors. The EFA yielded a six-factor model and converged with the CFA results, lending more credence to the obtained outcomes.

**Table 1:** Descriptive statistics and internal reliabilities (*n* = 155).

Variable	Mean	*SD*	Skewness	Kurtosis	Factor loadings	Cronbach’s alpha
General efficacy (GE)	4.13	1.10	−0.30	−0.75	0.79–0.89	0.88
Engagement efficacy (EE)	3.60	1.04	−0.19	−0.57	0.77–0.87	0.88
Interdisciplinary communication (IC)	4.01	0.91	−0.02	−0.43	0.64–0.86	0.90
Epistemic fluency (EF)	3.74	0.96	−0.21	−0.39	0.78–0.80	0.91
Technological pedagogical engineering knowledge (TPEK)	3.69	0.88	−0.24	0.02	0.71–0.81	0.89
Integrative STEM (iSTEM)	3.81	1.06	−0.19	−0.45	0.82–0.87	0.94

### Results of CFA Analyses

The CFA was performed using maximum likelihood estimation with data from the remaining participants (*n* = 328). The values of the item loading, average variance extracted (AVE), and composite reliability (CR) were computed to evaluate the convergent validity of the constructs (Hair et al., 2006). As shown in Table 2, all loading values of measured items are significant at *p* < 0.001 and greater than 0.7, revealing that more than 50% of the variance is explained by the constructs. Besides, the AVE and CR values were as: general efficacy (AVE = 0.60, CR = 0.86), engagement efficacy (AVE = 0.58, CR = 0.84), interdisciplinary communication (AVE = 0.50, CR = 0.85), epistemic fluency (AVE = 0.57, CR = 0.89), TPEK (AVE = 0.51, CR = 0.86), and iSTEM (AVE = 0.67. CR = 0.91). These values exceeded the cutoff values of 0.50 and 0.70, respectively. The fit for this model was good: χ2*/df* = 1.03; RMSEA = 0.009; SRMR = 0.04; CFI =0.99; and TLI = 0.99. CFI and TLI values equal to or larger than 0.95, and RMSEA and SRMR values equal to or smaller than 0.05 were obtained and indicate a good model fit (Hair et al., 2006). CFA analyses indicated an acceptable fit to the data, indicating the appropriateness of the instrument.

**Table 2:** The correlation matrix for the survey (*n* = 328).

	1	2	3	4	5	6
1. General efficacy (GE)	(0.77)					
2. Engagement efficacy (EE)	0.37***	(0.76)				
3. Interdisciplinary communication (IC)	0.14*	0.33***	(0.70)			
4. Epistemic fluency (EF)	0.40***	0.34***	0.52***	(0.75)		
5. Technological pedagogical engineering knowledge (TPEK)	0.34***	0.35***	0.44***	0.44***	(0.71)	
6. Integrative STEM (iSTEM)	0.39***	0.33***	0.55***	0.58***	0.38***	(0.81)

*Diagonal elements are the square roots of the AVE; off-diagonal elements are the correlation estimates*.

* *p < 0.05;*

*** *p < 0.001.*

Table 2 also presents the correlation matrix and the values of the square root of the AVE of each variable. According to Table 2, the square root of the AVE values of each construct is higher than 0.5 (Fornell and Larcker, 1981) and larger than the correlation coefficients between the construct and the others in the model (Chin, 1998). The convergent validity and discriminant validity of the proposed model are thus confirmed (Hair et al., 2006).

### SEM Analysis

**Figure 2:**
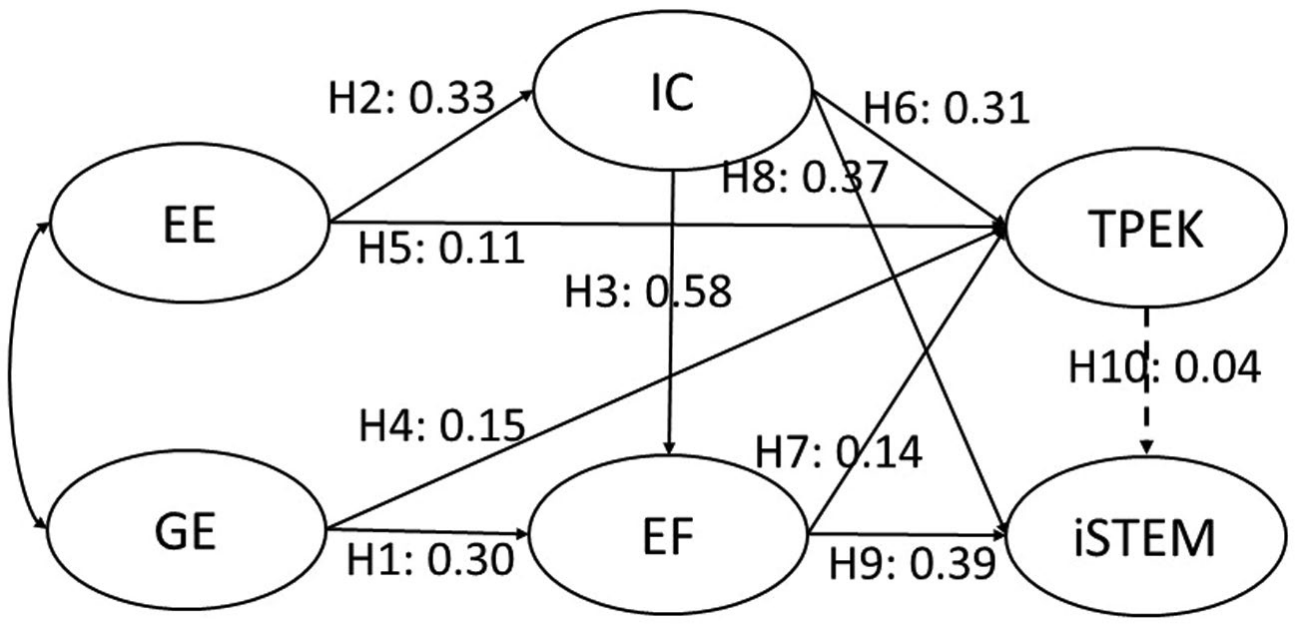
The structural model of the measured variables.

Path analysis was performed to test the hypothesized model. Ten hypotheses were tested through SEM. This path model demonstrated good fit: χ2*/df* = 1.03; RMSEA = 0.01; SRMR = 0.04; TLI = 0.99; and CFI = 0.99. Based on these indices, the model is acceptable (Hair et al., 2006). Nine out of 10 hypotheses were supported. The results obtained for the proposed hypotheses are presented in Table 3; Figure 2. The teachers’ general efficacy and interdisciplinary communication are both significant predictors of epistemic fluency (path coefficient = 0.30 and 0.58, *p* < 0.001) and engagement efficacy can predict interdisciplinary communication (path coefficient = 0.33, *p* < 0.001). The teachers’ general (path coefficient = 0.15, *p* < 0.01) and engagement (path coefficient = 0.11, *p* < 0.05) efficacies, interdisciplinary communication (path coefficient = 0.31, *p* < 0.001), and epistemic fluency (path coefficient = 0.14, *p* < 0.05) can predict TPEK. The teachers’ interdisciplinary communication (path coefficient = 0.37, *p* < 0.001) and epistemic fluency (path coefficient = 0.39, *p* < 0.001) can predict iSTEM; however, the teachers’ TPEK did not significantly predict iSTEM (path coefficient = 0.04, *p* = 0.47). The results indicated that the teachers’ TPEK did not show direct influence on their integrative STEM.

**Table 3:** Hypothesis testing.

Hypothesis		Estimate	*S.E.*	*C.R.*	*p* value	Supported Yes/No
H1	GE → EF	0.30	0.04	7.19	***	Yes
H2	EE → IC	0.33	0.05	6.24	***	Yes
H3	IC → EF	0.58	0.06	9.47	***	Yes
H4	GE → TPEK	0.15	0.05	3.19	**	Yes
H5	EE → TPEK	0.11	0.05	2.04	*	Yes
H6	IC → TPEK	0.31	0.07	4.43	***	Yes
H7	EF → TPEK	0.14	0.06	2.22	*	Yes
H8	IC → iSTEM	0.37	0.07	5.60	***	Yes
H9	EF → iSTEM	0.39	0.06	6.80	***	Yes
H10	TPEK → iSTEM	0.04	0.06	0.72	0.47	No

*GE: General efficacies, EE: Engagement efficacies, IC: interdisciplinary communication, EF: epistemic fluency, TPEK: technological pedagogical engineering knowledge, iSTEM: integrative STEM*.

* *p < 0.05;*

** *p < 0.01;*

*** *p < 0.001*.

### Measurement Invariance Across Different Groups

#### Invariance Tests Between Gender Groups

In a multi-group analysis of invariance, we began by testing the baseline model, which is also known as the configural model, to see if the structural patterns were identical for both gender groups (Hu and Bentler, 1999). The model showed a χ2 value of 900.905 (χ2/*df* = 1.065), TLI of 0.991, CFI of 0.992, and an RMSEA value of 0.014. Next, a metric invariance test was performed, where the factor pattern coefficients were constrained to be equal across the two gender groups. A good model fit showed a χ2 value of 918.329 (χ2/*df* = 1.054), TLI of 0.992, CFI of 0.993, and an RMSEA value of 0.013. Finally, a scalar invariance test was performed by constraining the intercepts to be the same across the two gender groups. The model fit indices showed an χ2 value of 962.933 (χ2/*df*  = 1.068), TLI of 0.991, CFI of 0.991, and an RMSEA value of 0.014. The related statistics are shown in Table 4.

**Table 4:** Fit indices for invariance tests.

		χ2	χ2/df	*p*	TLI	CFI	RMSEA
Gender	Configural invariance	900.905	1.065	0.093	0.991	0.992	0.014
	Metric invariance	918.329	1.054	0.129	0.992	0.993	0.013
	Scalar invariance	962.933	1.068	0.078	0.991	0.991	0.014
Subject matter	Configural invariance	1573.134	1.146	0.000	0.97	0.97	0.021
	Metric invariance	1599.534	1.144	0.000	0.97	0.97	0.021
	Scalar invariance	1631.913	1.142	0.000	0.971	0.97	0.021

#### Invariance Tests Among Subject Matter Groups

It is essential to test the invariance of the measurement model before conducting the latent mean analysis. First, configural invariance was assessed to determine the structural relationships in the theoretical model. The model showed that χ2 = 1573.134 (χ2/*df* = 1.146), TLI = 0.97, CFI = 0.97, and RMSEA = 0.021. Next, a metric invariance test was performed by constraining the factor pattern coefficients to be equal. A good model fit showed that χ2 = 1599.534 (χ2/df = 1.144), TLI = 0.97, CFI = 0.97, and RMSEA = 0.021. Finally, a scalar invariance test was performed by constraining the intercepts of the indicators to be the same. The results demonstrated a good model fit: χ2 = 1631.913 (χ2/*df*  = 1.142), TLI = 0.971, CFI = 0.97, and RMSEA = 0.021. The related statistics are shown in Table 4.

However, the χ2 test is sensitive to sample size. Cheung and Rensvold (2002) suggested that the absolute value of the confirmatory fit index (CFI) not greater than 0.01 be used as an evaluating criterion. Consequently, the analysis of the measurement invariance across gender and subject matters was validated, so latent mean analyses could be conducted.

#### Latent Mean Comparisons

To estimate the latent mean differences between the genders and subject matters concerning the dimensions in the scale, a group was selected as the reference group (i.e., the male group and ICT group, respectively). The latent mean of the dimensions for the reference group was set as 0, whereas that of the other groups was freely estimated.

In the gender group comparison, the latent mean represented a good model fit (χ 2 = 1015.493, *df* = 896, χ2 /*df* = 1.133, TLI = 0.981, CFI = 0.982, RMSEA = 0.020). In the subject matters group comparison, the latent mean represented a good model fit (χ2 = 1658.972, *df* = 1,402, χ2/*df* = 1.183, TLI = 0.962, CFI = 0.962, RMSEA = 0.024). Table 5 presents the results of the latent variables with no statistical difference in genders and subject matters. Overall, the results indicate that teachers’ general efficacies, engagement efficacies, interdisciplinary communication, epistemic fluency, TPEK, and integrative STEM capacities were not affected by gender or subject matters.

**Table 5:** Mean differences in gender and subject matters

	Gender (Female - Male)		Subject (Science - ICT)			Subject (Math - ICT)			
	Differences in latent mean	C.R.	P value	Differences in latent mean	C.R.	P value	Differences in latent mean	C.R.	P value
GE	−0.19	−1.23	0.22	−0.26	−1.59	0.11	0.02	0.11	0.91
EE	−0.23	−1.86	0.06	0.05	0.36	0.72	0.18	1.20	0.23
EF	0.07	0.58	0.56	0.19	1.41	0.16	0.14	0.89	0.37
IC	0.08	0.79	0.43	0.12	1.06	0.29	0.06	0.48	0.63
TEPK	−0.12	−1.38	0.17	0.13	1.40	0.16	0.15	1.36	0.17
iSTEM	0.13	1.18	0.24	0.06	0.46	0.65	0.14	0.92	0.36

GE: general efficacies, EE: engagement efficacies, IC: interdisciplinary communication, EF: epistemic fluency, TPEK: technological pedagogical engineering knowledge, iSTEM: integrative STEM.

## Discussion and Conclusion

Engineering design activities are springing up in K-12 education (Moore et al., 2014; Capobianco et al., 2018; Aranda et al., 2020). Designing integrative STEM instruction to engage students in STEM activities and to gain STEM-related thinking and problem-solving abilities are poised as crucial learning objectives of engineering and technology education (Kelley and Knowles, 2016; Hoeg and Bencze, 2017). When teachers design interdisciplinary STEM education, they are involved in multiple forms of knowledge and ways of knowing (Chai, 2019). Teachers need to integrate across different STEM disciplines to design purposeful STEM topics and activities. Nonetheless, in practice, there is limited research that unfolds teachers’ competencies (measured as efficacies in this study) needed to build their capacities for the teaching of integrated STEM curricula. In the present study, an instrument was designed to assess associated teachers’ STEM efficacies. Building on earlier studies of teachers’ efficacy (Tschannen-Moran et al., 1998; Künsting et al., 2016; Fackler et al., 2021) and teachers’ TPACK for STEM teaching (Chai et al., 2019), this study further identified teachers’ epistemic fluency and interdisciplinary communication (Markauskaite and Goodyear, 2017) as important competencies that teachers need for successful integrative STEM teaching. To this end, an instrument was built and validated with robust psychometric properties for future research. This study, therefore, contributes to the current STEM research by providing a good measurement tool for interested teacher educators. Future research could employ different professional development models and activities (see Chai, 2019) and measure the before and after developmental growth in relevant competencies.

The teachers’ GE and EE are positively correlated to their EF and IC. More importantly, the SEM indicated that GE and EE are predictive of teachers’ EF and IC. This provides support for our hypotheses that EF and IC are more advanced forms of competencies based on previous studies (Fackler et al., 2021; Lin et al., 2021; Pressley and Ha, 2021).

In particular, this study observed that the essential elements that could predict teachers’ efficacy for iSTEM are teachers’ epistemic fluency and interdisciplinary communication. This is generally aligned with previous studies (Boschman et al., 2015; Chiu et al., 2021). Nonetheless, these two forms of efficacy have been overlooked in previous studies.

Teachers’ epistemic fluency is acquired through interdisciplinary communication and collaborative knowledge work across disciplines. Studies have also indicated that STEM teachers’ epistemic fluency is a representation of their ability to represent and share ideas about professional knowledge, create new understanding, and take the perspectives of different epistemologies to integrate STEM knowledge (Morrison and Collins, 1996; Chai, 2019; Reynante et al., 2020). The SEM provided support that the teachers’ EF and IC are predictors of teachers’ TPEK and iSTEM efficacies.

Overall, the SEM indicated that teachers’ self-efficacy in teaching (i.e., GE and EE) could be viewed as the foundations for the development of more advanced competencies. The inclusion of EF and IC implies the processes that teachers need to be engaged in, which current literature suggests to be involving teachers in a community of practice for interdisciplinary collaborative design (Jho et al., 2016; Chai et al., 2020; Lin et al., 2021). With community-based professional development, teachers are likely to develop the competencies needed for iSTEM.

As discussed in the literature review, technology is integral to the implementation of today’s STEM curriculum. It performs multiple roles including as the platform for instruction delivery, providing cognitive tools for research, ideation, modeling, and communication (de Jong, 2019; Lee et al., 2020; Wang and Chiang, 2020). The factor TPEK refers to teachers’ efficacy in using technology to facilitate students’ learning of engineering knowledge. Theoretically, teachers’ TPEK should contribute to their iSTEM efficacy as indicated in previous research (Chai et al., 2019). In this study, TPEK was found to be not predictive of the teachers’ iSTEM, although they were positively correlated. One possible explanation for this finding could be that the training program has overemphasized the programming skills associated with Arduino. Other technological pedagogical knowledge needed such as the use of technology as cognitive tools (Lee, 2018) to foster integrative STEM efficacy has been overlooked. As computational skills are highly emphasized and widely promoted in today’s STEM education, the finding could serve to remind researchers and practitioners to avoid overfocusing on the computational aspects of STEM education. The teachers’ TPEK is conceptually richer than just coding. Teachers also need to learn how to select and present authentic problems that can engage students and facilitate students’ knowledge construction with technologies.

Regarding multi-group analyses across genders and subject matters, the configural, metric, and scalar tests attempt to establish the equivalence of the structure across genders and subject matters. The results of configural and metric invariance indicate that the factor loading pattern, factor loadings, and the structural weights appeared to be equivalent across the genders and subject matters examined. This suggests that this scale might be used to make comparisons between samples gathered in the STEM domain.

## Limitations

This study has some limitations. First, the sample was collected in China and the teachers have attended training for STEM instruction. However, as pointed out in the discussion, the training was overly focused on the coding of Arduino. Future studies may collect data from participants with different profiles with reference to the training or professional development the teachers received, and with participants from different countries and cultures. Second, it was a self-reported assessment. We attempted to assess teachers’ perceptions during iSTEM design processes, but the iSTEM design situations or conditions may vary in different contexts, and the training times, materials used, and the focus on professional development may have an impact on teachers’ design of integrated STEM (Yang et al., 2020; Chen and Terada, 2021). Despite these limitations, the findings of this study contribute to the literature by identifying STEM teachers’ efficacies, interdisciplinary collaboration, epistemic fluency, and professional knowledge for designing iSTEM learning.

## Data Availability Statement

The raw data supporting the conclusions of this article will be made available by the authors, without undue reservation.

## Author Contributions

P-YL: writing-original draft preparation, formal analysis, and validation. CC: conceptualization, methodology, and review and editing. XW: investigation, data curation, and resources. WD: project administration and funding acquisition. All authors have read and agreed to the published version of the manuscript.

## Funding

This research was funded by China University of Petroleum (East China) Postgraduate Innovation Project, grant number YCX2020098. Project supported by Open Research Fund of College of Teacher Education, Zhejiang Normal University (Grant No. JYKF22008).

## Conflict of Interest

The authors declare that the research was conducted in the absence of any commercial or financial relationships that could be construed as a potential conflict of interest.

## Publisher’s Note

All claims expressed in this article are solely those of the authors and do not necessarily represent those of their affiliated organizations, or those of the publisher, the editors and the reviewers. Any product that may be evaluated in this article, or claim that may be made by its manufacturer, is not guaranteed or endorsed by the publisher.

## Appendix

**Appendix 1:** The survey items

No.	Item
General Teaching Efficacy	
1	I am able to use different evaluation strategies to assess students' learning.
2	I am able to provide alternative explanations or examples to resolve students' doubts.
3	I can guide students' learning with good questions.
4	I am able to use different teaching strategies in my classroom.
Engagement Efficacy	
1	I can help my students to think critically
2	I can motivate students who show low interest in schoolwork
3	I can help my students to value learning
4	I can foster students' creativity
Epistemic fluency	
1	I am able to use different discipline-based methods of knowledge construction (e.g., science, mathematics, and engineering) flexibly
2	I can synthetically employ different ways of knowing (e.g., scientific reasoning, mathematical modeling, and design thinking) to resolve challenges that I face
3	I am well-versed in using different types of discipline-based thinking (e.g., scientific, computational, or design-oriented) to analyze the problems I need to solve
4	I am able to quickly combine different subject matter knowledge to contribute solutions to problems
5	I can easily adopt different disciplinary-based perspectives to understand emerging problems in society
6	I am able to synthesize different sources of information smoothly to analyze a problem from multiple perspectives
Interdisciplinary Communication	
1	I can communicate with colleagues from other disciplines in the context of interdisciplinary cooperation
2	I am able to transform different demands of interdisciplinary work to authentic learning opportunities through negotiation
3	I can deliberate matters pertaining to the integration of disciplines
4	I can explain my ideas to colleagues from other disciplines in a way they can understand
5	I am happy to listen to and accept solutions proposed by colleagues from other disciplines
6	I am confident that I can communicate smoothly with colleagues from other disciplines
TPEK	
1	I am able to present real-world problems using technology (video-cases, web site creators, etc.) to initiate an engineering project
2	I can engage students in building knowledge about the engineering design process using various forms of digital technologies (e.g., PowerPoint presentations, online videos)
3	I am competent in facilitating students' learning of various software tools that engineers use to develop their ideas (e.g., Computer-assisted design tools)
4	I can use technologies to scaffold students in solving complex engineering problems
5	I can use simulated environments to help students test their engineering design (e.g., bridge building simulator)
6	I can stimulate students' brainstorming of ideas for the engineering challenges with technology (e.g., mindmapping tools)
Integrative STEM	
1	I can design lessons that appropriately integrate interdisciplinary STEM content knowledge for student-centered learning
2	I am able to formulate good STEM problems to stimulate students’ interdisciplinary knowledge construction
3	I can plan complementary teaching and learning activities for the different subjects involved in STEM projects
4	I can facilitate students' interdisciplinary knowledge construction for the STEM projects using different ICT tools
5	I can help students build knowledge about how the STEM subjects are interconnected
