# Supplementary material for: Modeling Chinese Teachers’ Efficacies for the Teaching of Integrated STEM With Interdisciplinary Communication and Epistemic Fluency
Source: Front Psychol. 2022 Jun 2;13:908421. doi: 10.3389/fpsyg.2022.908421 (PMC9202477; doi:10.3389/fpsyg.2022.908421)
Supplement: Supplementary file 1 [file Data_Sheet_1.docx]

Supplementary Material

# Supplementary Figures and Tables

## Supplementary Figures


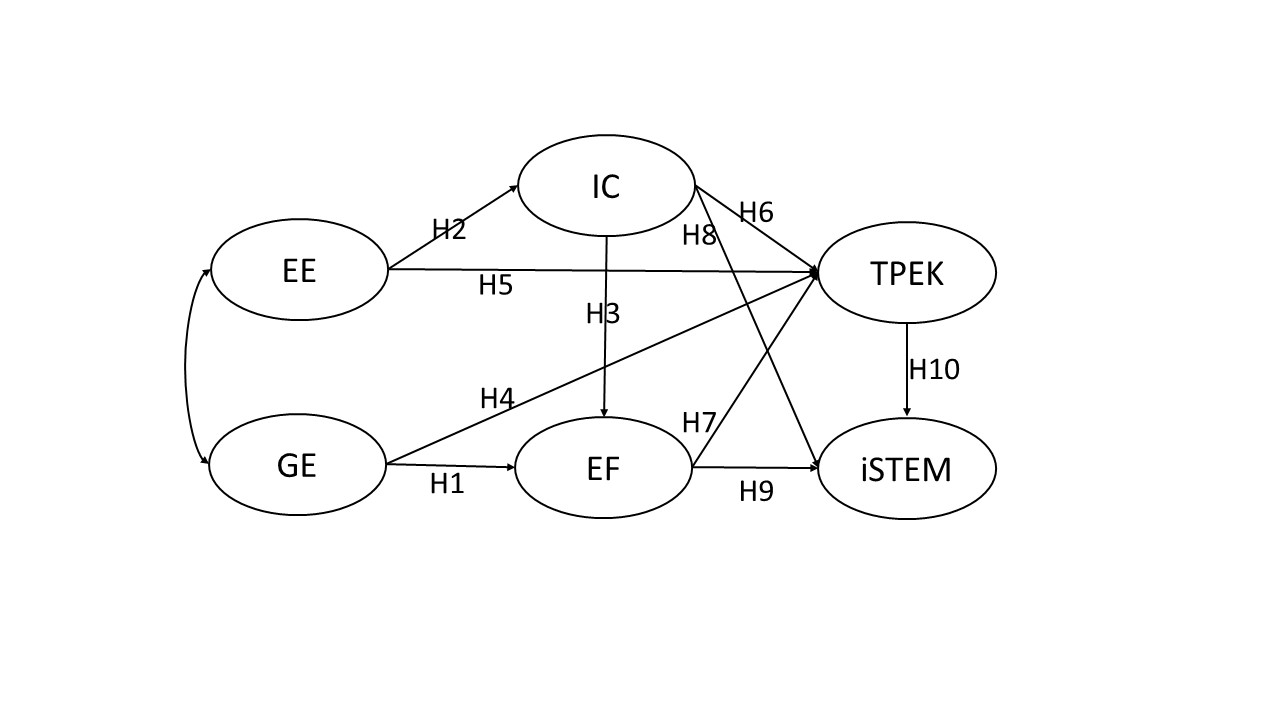


Figure 1. The hypothesized model

**
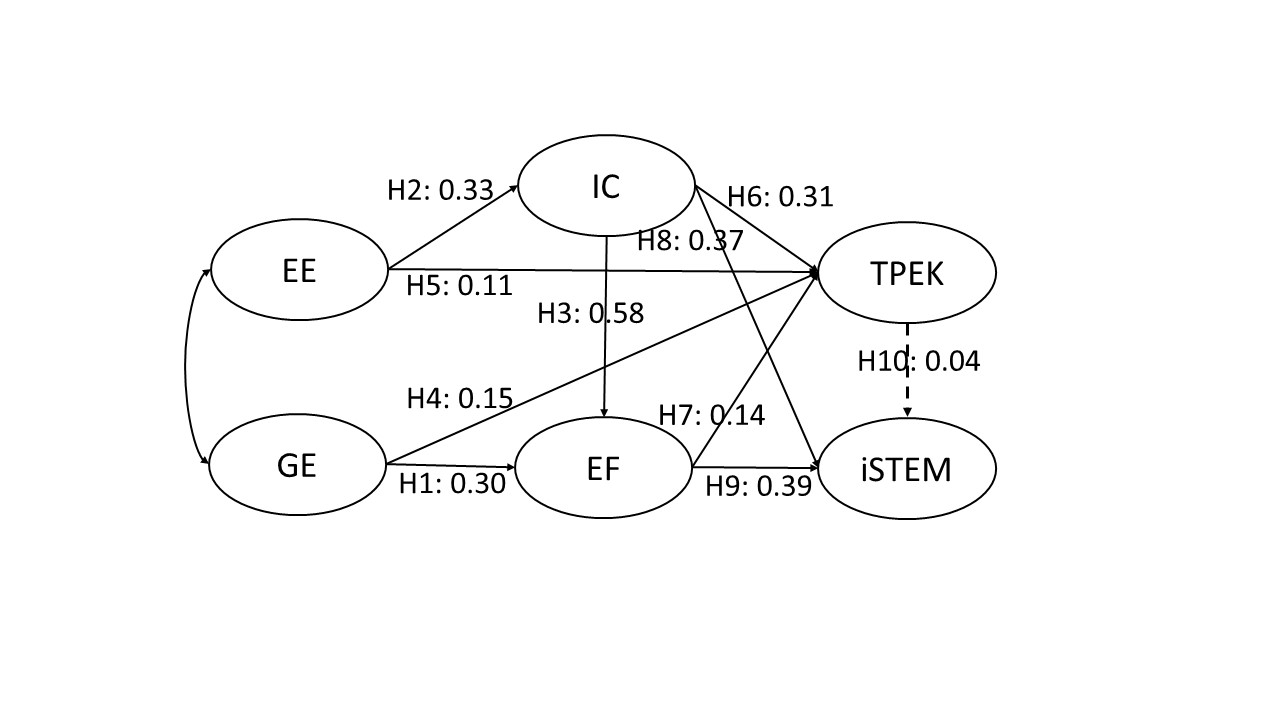
**

Figure 2. The structural model of the measured variables

## Supplementary Tables

Table 1. Descriptive statistics and internal reliabilities (*n* = 155)

| Variable | Mean | *SD* | Skewness | Kurtosis | Factor loadings | Cronbach’s alpha |
| --- | --- | --- | --- | --- | --- | --- |
| General efficacy (GE) | 4.13 | 1.10 | -0.30 | -0.75 | 0.79 – 0.89 | 0.88 |
| Engagement efficacy (EE) | 3.60 | 1.04 | -0.19 | -0.57 | 0.77 – 0.87 | 0.88 |
| Interdisciplinary communication (IC) | 4.01 | 0.91 | -0.02 | -0.43 | 0.64 – 0.86 | 0.90 |
| Epistemic fluency (EF) | 3.74 | 0.96 | -0.21 | -0.39 | 0.78 – 0.80 | 0.91 |
| Technological pedagogical engineering knowledge (TPEK) | 3.69 | 0.88 | -0.24 | 0.02 | 0.71 –0.81 | 0.89 |
| Integrative STEM (iSTEM) | 3.81 | 1.06 | -0.19 | -0.45 | 0.82 – 0.87 | 0.94 |

Table 2. The correlation matrix for the survey (*n* = 328)

|  | 1 | 2 | 3 | 4 | 5 | 6 |
| --- | --- | --- | --- | --- | --- | --- |
| 1. General efficacy (GE) | (0.77) |  |  |  |  |  |
| 2. Engagement efficacy (EE) | 0.37*** | (0.76) |  |  |  |  |
| 3. Interdisciplinary communication (IC) | 0.14* | 0.33*** | (0.70) |  |  |  |
| 4. Epistemic fluency (EF) | 0.40*** | 0.34*** | 0.52*** | (0.75) |  |  |
| 5. Technological pedagogical engineering knowledge (TPEK) | 0.34*** | 0.35*** | 0.44*** | 0.44*** | (0.71) |  |
| 6. Integrative STEM (iSTEM) | 0.39*** | 0.33*** | 0.55*** | 0.58*** | 0.38*** | (0.81) |

*Note*. * *p* < 0.05; *** *p* < 0.001

*Diagonal elements are the square roots of the AVE; off-diagonal elements are the correlation estimates.*

Table 3 Hypothesis testing

| Hypothesis | | Estimate | *S.E.* | *C.R.* | *P* value | Supported Yes/No |
| --- | --- | --- | --- | --- | --- | --- |
| H1 | GE → EF | 0.30 | 0.04 | 7.19 | *** | Yes |
| H2 | EE → IC | 0.33 | 0.05 | 6.24 | *** | Yes |
| H3 | IC → EF | 0.58 | 0.06 | 9.47 | *** | Yes |
| H4 | GE → TPEK | 0.15 | 0.05 | 3.19 | ** | Yes |
| H5 | EE → TPEK | 0.11 | 0.05 | 2.04 | * | Yes |
| H6 | IC → TPEK | 0.31 | 0.07 | 4.43 | *** | Yes |
| H7 | EF → TPEK | 0.14 | 0.06 | 2.22 | * | Yes |
| H8 | IC → iSTEM | 0.37 | 0.07 | 5.60 | *** | Yes |
| H9 | EF → iSTEM | 0.39 | 0.06 | 6.80 | *** | Yes |
| H10 | TPEK → iSTEM | 0.04 | 0.06 | 0.72 | 0.47 | No |

*Note*. * *p* < 0.05; ** *p* < 0.01; *** *p* < 0.001. GE: General efficacies, EE: Engagement efficacies, IC: interdisciplinary communication, EF: epistemic fluency, TPEK: technological pedagogical engineering knowledge, iSTEM: integrative STEM.

Table 4. Fit indices for invariance tests

|  | | χ2 | χ2/df | *p* | TLI | CFI | RMSEA |
| --- | --- | --- | --- | --- | --- | --- | --- |
| Gender | Configural invariance | 900.905 | 1.065 | 0.093 | 0.991 | 0.992 | 0.014 |
|  | Metric invariance | 918.329 | 1.054 | 0.129 | 0.992 | 0.993 | 0.013 |
|  | Scalar invariance | ​962.933 | 1.068 | 0.078 | 0.991 | 0.991 | 0.014 |
| Subject matter | Configural invariance | 1573.134 | 1.146 | .000 | 0.97 | 0.97 | 0.021 |
|  | Metric invariance | 1599.534 | 1.144 | .000 | 0.97 | 0.97 | 0.021 |
|  | Scalar invariance | 1631.913 | 1.142 | .000 | 0.971 | 0.97 | 0.021 |

Table 5. Mean differences in gender and subject matters

|  | Gender (Female - Male) | | | | Subject (Science - ICT) | | | | Subject (Math - ICT) | | | |
| --- | --- | --- | --- | --- | --- | --- | --- | --- | --- | --- | --- | --- |
|  | | Differences in latent mean | C.R. | P value | | Differences in latent mean | C.R. | P value | | Differences in latent mean | C.R. | P value |
| GE | | -0.19 | -1.23 | 0.22 | | -0.26 | -1.59 | 0.11 | | 0.02 | 0.11 | 0.91 |
| EE | | -0.23 | -1.86 | 0.06 | | 0.05 | 0.36 | 0.72 | | 0.18 | 1.20 | 0.23 |
| EF | | 0.07 | 0.58 | 0.56 | | 0.19 | 1.41 | 0.16 | | 0.14 | 0.89 | 0.37 |
| IC | | 0.08 | 0.79 | 0.43 | | 0.12 | 1.06 | 0.29 | | 0.06 | 0.48 | 0.63 |
| TEPK | | -0.12 | -1.38 | 0.17 | | 0.13 | 1.40 | 0.16 | | 0.15 | 1.36 | 0.17 |
| iSTEM | | 0.13 | 1.18 | 0.24 | | 0.06 | 0.46 | 0.65 | | 0.14 | 0.92 | 0.36 |

*Note*. GE: General efficacies, EE: Engagement efficacies, IC: interdisciplinary communication, EF: epistemic fluency, TPEK: technological pedagogical engineering knowledge, iSTEM: integrative STEM.
